# Male gynecomastia linked to antipsychotics: a case report

**DOI:** 10.1192/j.eurpsy.2024.1537

**Published:** 2024-08-27

**Authors:** O. Martin-Santiago, P. Martinez.Gimeno, M. Calvo-Valcarcel, C. Alario-Ruiz, B. Arribas-Simon

**Affiliations:** ^1^Hospital Clinico Universitario, Valladolid, Spain

## Abstract

**Introduction:**

Gynecomastia refers to the abnormal development of breast tissue in males, often posing a concerning symptom. Often, gynecomastia is associated with multiple factors, including the use of various drugs, notably certain atypical antipsychotics. Gynecomastia is a significant side effect that affects the quality of life of male patients taking antipsychotic medications. Among these, risperidone and paliperidone have been identified as the most prone to causing gynecomastia, although aripiprazole has garnered attention for its superior profile in controlling prolactin and gynecomastia. The relationship between these drugs and the development of gynecomastia lies in their ability to elevate prolactin levels, a hormone that regulates reproductive function and is involved in milk production. Several studies have shown that prolactin levels are more commonly elevated with risperidone and paliperidone prescription, thus triggering gynecomastia.

**Objectives:**

The study aims to investigate the management of gynecomastia in male patients receiving antipsychotic medications.

**Methods:**

This research employs a retrospective analysis of patient records to examine the association between specific antipsychotic drugs, prolactin levels, and the development of gynecomastia, while also evaluating the effectiveness of aripiprazole as an alternative treatment.

**Results:**

We present the case of a 21-year-old male with no prior medical history who initiated treatment with oral paliperidone and later switched to 100 mg of long-acting injectable paliperidone once monthly during his initial admission for psychotic symptoms. After six months, he developed gynecomastia, which was ruled out as breast tissue and was determined to be an increase in adipose tissue. Since his hospital discharge, he has gained 25 kg (30%) in body weight, and his baseline prolactin level has decreased. This weight gain, a common side effect of several antipsychotics, was linked to gynecomastia. However, a promising approach for gynecomastia antipsychotic-associated treatment is aripiprazole, which has a milder impact on prolactin levels. In this case, during the next appointment, a switch to 400 mg of long-acting injectable aripiprazole once-monthly was made, which led to weight loss, a reduction in breast size and blood prolactin levels in the following weeks.

**Conclusions:**

The detection and management of gynecomastia in these patients are crucial to improving their quality of life and treatment adherence. This management encompasses changes in medication, hormonal therapy, or surgery in severe cases. Physicians must be aware of this potential complication when prescribing antipsychotics and closely monitor at-risk patients. In summary, antipsychotic-associated gynecomastia in men represents a medical challenge that requires careful attention and an individualized treatment strategy for each affected patient.

**Disclosure of Interest:**

None Declared

